# Regulatory elements involved in the expression of competence genes in naturally transformable *Vibrio cholerae*

**DOI:** 10.1186/s12866-014-0327-y

**Published:** 2014-12-24

**Authors:** Mirella Lo Scrudato, Sandrine Borgeaud, Melanie Blokesch

**Affiliations:** Laboratory of Molecular Microbiology, Global Health Institute, School of Life Sciences, Swiss Federal Institute of Technology Lausanne (Ecole Polytechnique Fédérale de Lausanne, EPFL), EPFL-SV-UPBLO / Station 19, CH-1015 Lausanne, Switzerland

**Keywords:** Natural competence, Transformation, *Vibrio cholerae*, cAMP receptor protein, Quorum sensing

## Abstract

**Background:**

The human pathogen *Vibrio cholerae* normally enters the developmental program of natural competence for transformation after colonizing chitinous surfaces. Natural competence is regulated by at least three pathways in this organism: chitin sensing/degradation, quorum sensing and carbon catabolite repression (CCR). The cyclic adenosine monophosphate (cAMP) receptor protein CRP, which is the global regulator of CCR, binds to regulatory DNA elements called CRP sites when in complex with cAMP. Previous studies in *Haemophilus influenzae* suggested that the CRP protein binds competence-specific CRP-S sites under competence-inducing conditions, most likely in concert with the master regulator of transformation Sxy/TfoX.

**Results:**

In this study, we investigated the regulation of the competence genes *qstR* and *comEA* as an example of the complex process that controls competence gene activation in *V. cholerae*. We identified previously unrecognized putative CRP-S sites upstream of both genes. Deletion of these motifs significantly impaired natural transformability. Moreover, site-directed mutagenesis of these sites resulted in altered gene expression. This altered gene expression also correlated directly with protein levels, bacterial capacity for DNA uptake, and natural transformability.

**Conclusions:**

Based on the data provided in this study we suggest that the identified sites are important for the expression of the competence genes *qstR* and *comEA* and therefore for natural transformability of *V. cholerae* even though the motifs might not reflect *bona fide* CRP-S sites.

## Background

*Vibrio cholerae* is a Gram-negative bacterium that often lives in aquatic environments in association with the chitinous exoskeleton of zooplankton [1,2]. Chitin, a polymer of β-1,4-linked N-acetylglucosamine, is one of the most abundant biopolymers in nature [3]. In addition to its role as a nutrient source, chitin also induces natural competence for transformation in *V. cholerae* [4] and other *Vibrio* species (reviewed by [5]).

**Figure 1:**
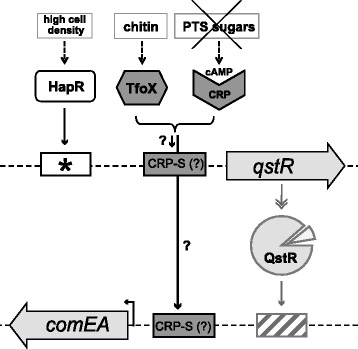
**Proposed working model describing the transcriptional regulation of the competence genes** ***qstR*** **and** ***comEA*** **within the network of natural competence for transformation of** ***V. cholerae*** **.** ***qstR*** **and** ***comEA*** **are tightly regulated by three different pathways: quorum sensing, chitin-sensing/degradation, and carbon catabolite repression (signals indicated on top; from left to right).** Upon growth on chitin or artifical induction, TfoX is produced within the cell. Furthermore, unsaturated PTS systems lead to the activation of the enzyme adenylate cyclase, which synthesizes cAMP. cAMP then forms a complex with CRP. Based on work performed in *H. influenzae* [20,21], we hypothesized that CRP-cAMP might bind to the putative CRP-S sites described here and that such binding would be dependent on TfoX. However, full expression of those genes requires a QS-dependent secondary activator (HapR and QstR, respectively). At high cell density the master regulator of QS, HapR, is produced and allows the expression of *qstR* by directly binding to its promoter (indicated by an asterisk marking a HapR binding site identified *in vitro*; [22]. The transcriptional regulator QstR, which might require a cofactor for its full activity (indicated by the triangle), positively regulates the *comEA* gene potentially by binding to a QstR-specific binding motif (indicated by the dashed box and so far unidentified). The question marks indicate the subjects addressed in this study.

Natural competence is a mode of horizontal gene transfer, which is based on the ability of a bacterium to take up free DNA from the environment and recombine it with the bacterial genome resulting in natural transformation. In *V. cholerae,* chitin leads to the up-regulation of *tfoX* [4,6] (Figure 1). This gene encodes a protein that is the master regulator of transformation and a homolog of Sxy, which was first described in *Haemophilus influenzae* [7,8]. Indeed, *tfoX* expression is sufficient to induce natural competence and transformation in *V. cholerae* even in the absence of chitin [4,9,10]. In our current working model, the components of a type IV pilus combined with a few other competence proteins (such as ComEA, ComEC, and ComF) make up the majority of the DNA-uptake machinery. This machinery is responsible for binding to and pulling extracellular DNA into the periplasm of *V. cholerae* and subsequently, into the cytoplasm [5,11-14] as previously suggested for other naturally competent bacteria (reviewed for example by [15-19]).

In addition to TfoX expression, pathways that regulate quorum sensing (QS) and carbon catabolite repression (CCR) are also necessary to induce the competence regulon of *V. cholerae* [11,23] (Figure 1). QS is a process of bacterial communication and is based on the production and secretion of small molecules called autoinducers (reviewed by [24]). *V. cholerae* produces and secretes at least two different autoinducers: the intra-species cholera autoinducer 1 (CAI-1) and the universal autoinducer 2 (AI-2) [24-28]. At high cell density, the concentration of autoinducers is sufficient to lead to the production of HapR, the master regulator of QS that is known to regulate virulence repression [25,29,30], biofilm formation [31] and natural competence for transformation [4,5,9-11,22,32-35] (Figure 1). In the absence of HapR, the extracellular DNA is degraded by the action of the nuclease Dns, preventing DNA uptake [9,11,33]. HapR regulates natural transformation by direct repression of *dns* and concomitantly with TfoX-mediated induction, directly drives the expression of *qstR*, which encodes the newly identified transcription factor QstR [22] (Figure 1). Notably, the contribution of QstR to natural transformation was recently confirmed by Dalia *et al*. using a genome-wide transposon sequencing (Tn-seq) screen [36].

The third pathway involved in the regulation of natural competence for transformation is CCR [4,23]. This term indicates the mechanism by which, in the presence of a preferred carbon source such as glucose, the expression of genes necessary for the metabolism of other carbon sources is repressed [37]. The major players in CCR are the phosphoenolpyruvate-carbohydrate phosphotransferase system (PTS), adenylate cyclase (CyaA), the metabolite 3′,5′-cyclic adenosine monophosphate (cAMP) and the CRP protein. Unsaturated PTS transporters enhance the synthesis of cAMP by CyaA. High levels of cAMP trigger the formation of an active complex of CRP and cAMP, which binds the promoters of the target genes (e.g., those genes encoding proteins that are involved in the transport and utilization of alternative carbon sources). Conversely, when the PTS is saturated cAMP is not produced and the CRP-cAMP complex cannot form. Central metabolism and transport of the carbon sources are not the exclusive targets of CCR; cAMP and the CRP protein, as well as the PTS components (independent of cAMP), also control biofilm formation in *V. cholerae* [38-40]. With respect to natural competence for transformation, the presence of PTS sugars significantly decreases the transformability of *V. cholerae*; moreover, knockout strains for *crp* or *cyaA* are non-transformable [23].

The role and function of the CRP protein have been primarily studied in *E. coli* (reviewed by [41,42]). CRP, formerly known as catabolite activator protein (CAP), forms a dimer of two identical subunits. Each CRP subunit contains an N-terminal cAMP binding domain, a flexible hinge region and a C-terminal helix-turn-helix DNA binding motif. CRP recognizes and binds 22 bp-long symmetrical sequences called CRP sites. Under physiological conditions, CRP is likely present either as a free apo-CRP dimer (in the absence of cAMP) or as a dimer with each subunit bound to a molecule of cAMP. *V. cholerae* CRP and *E. coli* CRP (EcCRP) share 95% identity in amino acid sequence [43]. As with EcCRP, *V. cholerae* CRP displayed a biphasic dependence on cAMP levels *in vitro*. Moreover *V. cholerae* CRP is able to activate the transcription of *E. coli gal* promoters [44,45]. These findings strongly suggest that the CRP protein functions similarly in these two bacterial species.

In *H. influenzae,* the expression of the competence genes requires the CRP-cAMP complex [46,47] along with the master regulator of transformation Sxy [7] (TfoX in *V. cholerae*). The competence regulon of *H. influenzae* consists of genes characterized by the presence of competence regulatory elements (CRE) [48,49]. Due to their Sxy dependency, these specific competence-related CRP binding sites were later renamed CRP-S sites to distinguish them from the canonical Sxy-independent CRP-N sites [20]. Indeed, Cameron and Redfield suggested that in *H. influenzae,* and most likely in other competent Gram-negative bacteria, the induction of competence genes is under the control of CRP and Sxy/TfoX acting in concert at the CRP-S site [20]. Based on previously published expression data from *V. cholerae* [4,6] Cameron and Redfield also predicted a 22-bp CRP-S and CRP-N consensus motif for the *Vibrionaceae* family *in silico*, which was nnntTTnAAnTnnnTCGAAnnn for CRP-S and tnntGTGAnnnnnnTCACanan for CRP-N (the most common bases are indicated in upper case, the less likely bases in lower case; 'n' refers to any base even though minor preference might be valid for some of these positions; for details see [20]).

In this study, we tested the role of QstR as an activator of *comEA*. We demonstrated that overexpression of *qstR* was sufficient to increase the abundance of the *comEA* transcripts, although not to the same level observed under competence-inducing conditions. We therefore hypothesized that TfoX and CRP-cAMP were also involved in driving the expression of *comEA* and *qstR* (Figure 1). In agreement with this hypothesis, we identified putative CRP-S sites in the promoter regions of *qstR* and *comEA,* which were not part of the *in silico* predicted *Vibrionaceae* CRP-S sites described above [20]. We investigated the importance of these motifs using site-directed mutagenesis, followed by the analysis of the respective mutants. Our results suggest that these sites are important for the transcriptional regulation of the respective competence genes but might not represent *bona fide* CRP-S sites.

## Methods

### Bacterial strains and plasmids

*Vibrio cholerae* strains and plasmids used in this study are listed in Table 1. *Escherichia coli* strains DH5α [50] and One Shot PIR1 or PIR2 (Invitrogen) were employed as hosts for cloning. *E. coli* strain S17-1λpir [51] was used as a mating donor for plasmid transfer between *E. coli* and *V. cholerae*.

**Table 1:** **Strains and plasmids used in this study**

**Strains or plasmids**	Genotype*/Description	**Reference**
***V. cholerae*** **strains**		
A1552	Wild-type, O1 El Tor Inaba, Rif^R^	[52]
A1552-LacZ-Kan	A1552 strain with *aph* cassette in *lacZ* gene; Rif^R^, Kan^R^	[53,54]
A1552-Tn*tfoX*	A1552 containing mini-Tn7-*araC*-P_*BAD*_-*tfoX*; Rif^R^, Gent^R^	[10]
ΔhapR	A1552ΔVC0583, Rif^R^	[4]
ΔhapR-Tn*tfoX*	A1552ΔhapR containing mini-Tn7-*araC*-P_*BAD*_-*tfoX*; Rif^R^, Gent^R^	[10]
ΔcomEA	A1552ΔVC1917 (=A1552*VC1917* in (Ref)), Rif^R^	[4]
ΔcomEA-Tn*tfoX*	A1552ΔcomEA containing mini-Tn7-*araC*-P_*BAD*_-*tfoX*; Rif^R^, Gent^R^	[22]
ΔqstR	A1552ΔVC0396, Rif^R^	[22]
ΔqstR-Tn*tfoX*	A1552ΔqstR containing mini-Tn7-*araC*-P_*BAD*_-*tfoX*; Rif^R^, Gent^R^	[22]
ΔCRP-S	CRP-S site upstream of *comEA* deleted in strain A1552-Tn*tfoX* using the TransFLP method; Rif^R^, Gent^R^	This study
CRP-S_inv	CRP-S site upstream of *comEA* inverted in strain A1552-Tn*tfoX* using the TransFLP method; Rif^R^, Gent^R^	This study
CRP-N	CRP-S site upstream of *comEA* changed for a CRP-N site (see scheme in Figure 4) in strain A1552-Tn*tfoX* using the TransFLP method; Rif^R^, Gent^R^	This study
[*frdA*] site	CRP-S site upstream of *comEA* changed for the *in silico* predicted CRP-N site preceding the *frdA* gene in strain A1552-Tn*tfoX* (see scheme in Figure 4) using the TransFLP method; Rif^R^, Gent^R^	This study
CRP-0	CRP-S site upstream of *comEA* changed in the 3′ conserved region (see scheme in Figure 4) in strain A1552-Tn*tfoX* using the TransFLP method; Rif^R^, Gent^R^	This study
WT_qstR (FRT control)	Extended TransFLP scar [53,55] added upstream of *qstR* without changing the CRP-S site (control) in strain A1552-Tn*tfoX*; Rif^R^, Gent^R^	This study
ΔHapR-site_qstR	HapR-binding site determined *in vitro* [22] deleted from strain A1552-Tn*tfoX* using the TransFLP method; Rif^R^, Gent^R^	This study
ΔCRP-S_qstR	CRP-S site upstream of *qstR* deleted in strain A1552-Tn*tfoX* (see scheme in Figure 4) using the TransFLP method; Rif^R^, Gent^R^	This study
[*frdA*] site_qstR	CRP-S site upstream of *qstR* changed for the *in silico* predicted CRP-N site preceding the *frdA* gene (see scheme in Figure 4) in strain A1552-Tn*tfoX* using the TransFLP method; Rif^R^, Gent^R^	This study
CRP-0_qstR	CRP-S site upstream of *qstR* changed in the 3′ conserved region (see scheme in Figure 4) in strain A1552-Tn*tfoX* using the TransFLP method; Rif^R^, Gent^R^	This study
**Plasmids**		
pBR322	Amp^R^, Tc^R^	[56]
pBAD/Myc-HisA	pBR322-derived expression vector; *araBAD* promoter (P_BAD_); Amp^R^	Invitrogen
p_*qstR*	*qstR* gene cloned into pBAD/Myc-HisA; arabinose inducible; Amp^R^	[22]
pUX-BF13	*ori*R6K, helper plasmid with Tn*7* transposition function; Amp^R^	[57]
pGP704::Tn7	pGP704 with mini-Tn7	[58]
pGP704-mTn7-*araC*-*tfoX*	pGP704 with mini-Tn7 carrying *araC* and P_*BAD*_-driven *tfoX;* Amp^R^	[10]
pBR-Tet_MCSI	pBR322 derivative deleted for Tet promoter and part of *tet* ^R^ gene; new MCS included; Amp^R^	[10]
pBR-Tet_MCSII	pBR322 derivative deleted for Tet promoter and part of *tet* ^R^ gene; new MCS included; Amp^R^	[22]
pBR-[own]*comEA*	*comEA* gene preceded by 900 bp of upstream region cloned into pBR-Tet_MCSII; Amp^R^	[22]
pBR-[−700]*comEA*	*comEA* gene preceded by 700 bp of upstream region; plasmid generated by inverse PCR of pBR-[own]*comEA*; Amp^R^	This study
pBR-[−500]*comEA*	*comEA* gene preceded by 500 bp of upstream region; plasmid generated by inverse PCR of pBR-[own]*comEA*; Amp^R^	This study
pBR-[−300]*comEA*	*comEA* gene preceded by 300 bp of upstream region; plasmid generated by inverse PCR of pBR-[own]*comEA*; Amp^R^	This study
pBR-[−134]*comEA*	*comEA* gene preceded by 134 bp of upstream region cloned into *Not*I site of pBR-Tet_MCSII; Amp^R^	This study
pBR-[−100]*comEA*	*comEA* gene preceded by 100 bp of upstream region; plasmid generated by inverse PCR of pBR-[own]*comEA*; Amp^R^	This study
pBR-[−40]*comEA*	*comEA* gene preceded by 40 bp of upstream region; plasmid generated by inverse PCR of pBR-[own]*comEA*; Amp^R^	This study

*VC numbers according to [59].

### Media and growth conditions

*V. cholerae* and *E. coli* strains were grown at either 30°C or at 37°C. Overnight cultures were grown in LB medium under aerobic conditions. Thiosulfate Citrate Bile Salts Sucrose (TCBS) agar plates were used to counterselect *E. coli* strains after triparental mating with *V. cholerae* strains. The TCBS agar plates were prepared following the manufacturer’s instructions (Fluka). For plasmid maintenance or selection of transformants/transconjugants, antibiotics were added to the growth media at concentrations of 50 or 100 μg ml^−1^ for ampicillin, 75 μg ml^−1^ for kanamycin, and 50 μg ml^−1^ for gentamicin.

### Construction of *V. cholerae* mutant strains

Chromosomally-encoded site-directed mutants were generated using the previously described TransFLP method [53,55,60]. The extended FRT scar was located downstream of the native *comEA* gene and upstream of the native *qstR* gene*.* In the latter case, a control strain was designed that was not modified in a site-directed manner but solely contained the integrated FRT scar upstream *qstR* (strain WT_qstR (FRT control)). This strain behaved as the WT with respect to the expression pattern, natural transformation, DNA uptake, and general growth behavior (described below).

### Construction of plasmids

The majority of the plasmids carrying the *comEA* gene preceded by a certain length of its upstream region were made through inverse PCR, using the plasmid pBR-[own]*comEA* [22] as template and the oligonucleotides listed in Table 2. The plasmid pBR-[−134]*comEA* was constructed by cloning the PCR-amplified insert [−134]*comEA* into the *Nco*I site of pBR-Tet_MCSII [22].

**Table 2:** **Primers used in this study**

**Primer name**	**Sequence**	**Description**
	**(in 5′ to 3′ direction)**	
Rev[VC1917]-NotI	GCGGCCGCGAGCTCTAGAGGTTTCTTAG	For inverse PCR leading to plasmids:
		pBR-[−700]*comEA*,
		pBR-[−500]*comEA*,
		pBR-[−300]*comEA*,
		pBR-[−100]*comEA*,
		pBR-[−40]*comEA*
Fwd[VC1917]-700	AGAGCTCGCGGCCGCAGGTGTTAACCACTCCTGCGGTAC	Inverse PCR to generate
		pBR-[−700]*comEA*
Fwd[VC1917]-500	AGAGCTCGCGGCCGCCAACAAGCACTTGAACTGGGTAAC	Inverse PCR to generate
		pBR-[−500]*comEA*
Fwd[VC1917]-300	AGAGCTCGCGGCCGCTATCGTTGTGATTGAGTTGAGC	Inverse PCR to generate
		pBR-[−300]*comEA*
VC1917-134-NotI	GCGGCGGCCGCATTCTTAGTGTAATTGATATG	PCR to generate
pBR-TET_MCS after	ATCATGCGCACCCGTGGCCAGGACCC	pBR[−134]*comEA*,
Fwd[VC1917]-100	AGAGCTCGCGGCCGCGGGCTACAGCAGTAGCCCGTTC	Inverse PCR to generate
		pBR[−100]*comEA*
Fwd[VC1917]-40	AGAGCTCGCGGCCGCCGCTATCATAAGCCCTCAACAAC	Inverse PCR to generate
		pBR[−40]*comEA*
gyrA-157-fwd	AATGTGCTGGGCAACGACTG	qRT-PCR for *gyrA* transcription [10]
gyrA_332_bwd	GAGCCAAAGTTACCTTGGCC	
comEA_50_fwd	CGACATTACCGTTACTGGCC	qRT-PCR for *comEA* transcription [10]
comEA_224_bwd	CCGTTGGCTTCTCGATAATCG	
comEC_1029_fwd	GGTCGCGATTGTTCTCTACC	qRT-PCR for *comEC* transcription [10]
comEC_1186_bwd	CCAAATTGTACAGAACTGCCG	
VC0396_188_fwd	GCCTGATTCGCCAGCAATTG	qRT-PCR for *qstR* transcription [22]
VC0396_356_bwd	CCAAGACCGTGGGCAATAAAG	
hapR-230-fwd	CCAACTTCTTGACCGATCAC	qRT-PCR for *hapR* transcription [22]
hapR-399-bwd	GGTGGAAACAAACAGTGGCC	
hapA_175_fwd	ACGGTACAGTTGCCGAATGG	qRT-PCR for *hapA* transcription [22]
hapA_358_bwd	GCTGGCTTTCAATGTCAGGG	
comEA_284_rev	CGCACTGTCGCTTCACCAATCC	5′RACE: synthesis of first strand cDNA of *comEA*
comEA_217_rev	CTTCTCGATAATCGACAATGGCCTGAGC	5′RACE: PCR amplification of Poly(A) cDNA *comEA*
oligo dT-Anchor primer (Roche)	GACCACGCGTATCGATGTCGAC	
F-EcoRI_Anchor_P	CCAAGAATTCGACCACGCGTATCGATGTCGAC	5′RACE: PCR fragment of Poly(A) cDNA *comEA* cloned into plasmid pBR-Tet_MCSI
R-BamHI_comEA_217	CCAAGGATCCCTTCTCGATAATCGACAATGGCCTGAGC	
T7RNA-pol-750-down	GCTGAGGCTATCGCAACCCGTGC	DNA uptake assay: amplification of donor DNA; primer specific for *E. coli* BL21(DE3); [9,13]
T7 RNAP-end-bw	TTACGCGAACGCGAAGTCCGACTCTAAG	
lacZ-missing-fw	GCCGACTTTCCAATGATCCACAATGGG	DNA uptake assay: amplification of acceptor DNA; primer specific for *V. cholerae* A1552 and lacZ^+^ derivatives of it; [9,13]
lacZ-missing-bw	CCCTCGCTATCCCATTTGGAAATGCC	

### Natural transformation assay (chitin-dependent and chitin-independent)

Natural transformation assays were performed as previously described, growing *V. cholerae* strains on chitin flakes with a medium change on day two [54], or in LB medium supplemented with 0.02% arabinose to express an inducible chromosomal copy of *tfoX* (preceeded by a P_BAD_ promoter; [10]). The same growth conditions were used for *in trans* over-expression of the plasmid-encoded *qstR* gene*.* Notably, *V. cholerae* does not contain any obvious homolog of the low-affinity high-capacity arabinose transporter AraE*,* which is involved in the all-or-none induction of genes preceeded by the arabinose-inducible promoter P_BAD_ in *E. coli* [61]. Statistical analyses of transformation data were carried out on log-transformed data [62] using a two-tailed Student’s *t*-test.

### Whole-cell duplex PCR assay to test for DNA uptake

DNA uptake was verified using a whole-cell duplex PCR assay as previously described [9,13]. Briefly, the respective *V. cholerae* strains were induced for competence as described above before genomic DNA of *E. coli* strain BL21(DE3) was added at a final concentration of 2 μg/ml. After a 2 h incubation step the cells were harvested and DNase I-treated. Any excess nuclease was removed by washing of the cells with PBS buffer. ~3×10^6^ bacteria served as template in a whole-cell duplex PCR. The two primer pairs were specific for the donor DNA derived from *E. coli* BL21(DE3) and for gDNA of the *V. cholerae* acceptor strain (at a 10-fold lower concentration), respectively [9,13].

### SDS-PAGE and Western blotting

Proteins were separated by sodium dodecyl sulfate (SDS)-polyacrylamide gel electrophoresis (SDS-PAGE) and then subjected to western blotting as previously described [10]. The primary antibody against ComEA (GP 1248; see below) and horseradish peroxidase (HRP)-conjugated goat anti-rabbit secondary antibody (Sigma-Aldrich, Switzerland) were diluted at 1:5,000 and 1:20,000, respectively. Luminescent signals were produced and detected by Western Lightning-ECL (PerkinElmer) and chemiluminescence-detecting film (Amersham Hyperfilm ECL, GE Healthcare).

### Generation of the antibodies against ComEA

Rabbit anti-ComEA antibodies were raised against synthetic peptides and produced by Eurogentec (Belgium). The antibody was tested in Western blot analysis against the *comEA* knockout strain to exclude potential cross-reactions with proteins migrating towards the same position as the target protein.

### Quantitative reverse transcription PCR (qRT-PCR)

*V. cholerae* strains were grown in LB medium supplemented with 0.02% arabinose to induce the *qstR* gene or natural competence. RNA preparation, DNase treatment, reverse transcription, and qPCR were performed as previously described [10,22,23].

### 5′ Rapid amplification of cDNA ends (5′RACE)

*V. cholerae* wild-type strain A1552-Tn*tfoX* was induced to competence as described above. Cell harvesting and RNA preparation were performed as previously published [22]. The 5′/3′ RACE Kit 2nd Generation (Roche) was used to identify the transcription start of the *comEA* gene. All steps were performed according to the manufacturer’s protocol unless stated otherwise. Total RNA (2 μg) and the gene-specific primer comEA_284_rev were used to synthesize the first strand cDNA of *comEA*. The cDNA was then purified using the High Pure PCR Product Purification Kit (Roche, Switzerland). After addition of the Poly(A) tail to the 3′ end, the first strand cDNA was amplified by PCR with the gene-specific primer comEA_217_rev and oligo dT-Anchor primer. The PCR products were visualized on an agarose gel and purified. The double-stranded cDNA of *comEA* was further amplified by PCR with the primers F-EcoRI_Anchor_P and R-BamHI_comEA_217. The PCR products were cloned into the *EcoR*I/*BamH*I sites of the plasmid pBR-Tet_MCSI [10]. To determine the transcription start point of *comEA*, fifteen of those plasmids were sequenced. 12 out of those 15 sequences pointed to the C at position −24 bp upstream the *comEA* start codon as transcriptional start site.

## Results

### Regulation of *comEA* by QstR and TfoX/CRP-cAMP

**Figure 2:**
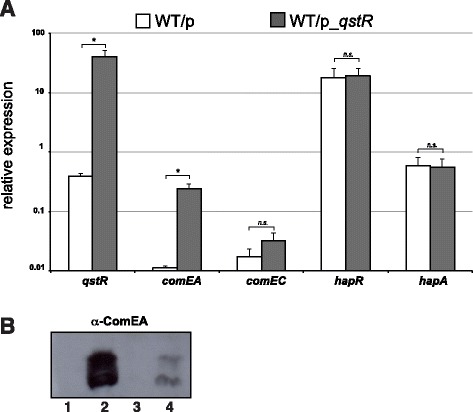
**QstR drives the expression of** ***comEA*** **in the absence of competence induction. (A)** qRT**-**PCR data showing the relative expression of the indicated genes in wild-type *V. cholerae* strain A1552 (WT) carrying an empty vector (p) as control or a plasmid encoding *qstR* (p_*qstR*; ara-inducible). Strains were grown to high cell density, and the growth medium was supplemented with arabinose to induce expression of *qstR* in the absence of competence-induction (e.g., independent of TfoX). Data are the average of three biological replicates. Error bars indicate standard deviation. Statistically significant differences were determined by Student’s *t-*tests*.* *P < 0.05, *n.s.* = not significant. **(B)** Detection of the ComEA protein (with and without the N-terminal signal sequence peptide; upper and lower band, respectively) by Western blot analysis using protein-specific antibodies. Total protein was extracted from the indicated strains after growth to high cell density. Lanes: 1, *V. cholerae* strain A1552-Tn*tfoX,* competence-non-induced; 2, A1552-Tn*tfoX* competence-induced; 3, wild-type strain A1552 carrying plasmid pBAD/Myc-HisA (vector control); 4, A1552 containing plasmid p_*qstR in trans*. Strains indicated in lanes 3 and 4 were grown in the presence of 0.02% arabinose to induce the P_BAD_ promoter, which is located on the plasmid.

From previous studies it was known that the expression of *comEA* is dependent on *a)* the master regulator of transformation, TfoX; *b)* the CRP-cAMP complex; and *c)* the transcription factor QstR [4,22,23] (Figure 1). Indeed, we previously demonstrated that the QstR protein is required for proper expression of the two competence genes, *comEA* and *comEC* [22], both of which are essential for DNA uptake and natural transformation [12]. We also speculated that QstR might require a cofactor for proper binding to the respective promoter regions [22]. Here, we asked whether TfoX only regulates *comEA* indirectly through its influence on QstR, or whether it also has a role in directly regulating the expression of *comEA* (Figure 1). To do so, we measured the transcripts of *comEA* in the absence of natural competence induction (e.g., in the absence of a chitin surface and without artificial induction of the gene encoding the main regulator of transformation, TfoX) but with *in trans* overexpression of *qstR*. Using this approach, we observed an increased level of *comEA* expression, whereas expression of the upstream acting regulatory gene *hapR* and the competence-unrelated but HapR-activated gene *hapA* did not change as expected (Figure 2). However, the relative expression of *comEA* appeared lower than what was measured when natural competence was concomitantly induced even though the expression of *qstR* was significantly higher due to the multi-copy effect of the expression *in trans* (compare to [22] and data shown below).

Next, we investigated the production of the ComEA protein in order to evaluate whether the transcript levels would also reflect the protein levels. Apart from the strains used to measure the *comEA* transcripts, we also included wild-type strains harboring an inducible copy of *tfoX* on the chromosome as previously described [10] and grew the strains under both competence-inducing and non-inducing conditions. Total protein extracts were prepared from bacterial strains that had reached the high cell density state. As shown in Figure 2B, the ComEA protein was readily detectable in the competence-induced strain (lane 2) and absent in the same strain that was grown without competence induction (lane 1). Moreover, we detected low levels of ComEA in the wild-type strain when *qstR* was overexpressed but competence (e.g., *tfoX*) was not induced (lane 4), which was not the case for the vector control (lane 3). This result strengthened the evidence that QstR *per se* is able to drive the expression of *comEA* in the absence of competence induction. However, the protein was more abundant in the wild-type strain under competence-inducing conditions, suggesting that full *comEA* expression requires more than the QstR protein alone, which is consistent with the proposed dual regulation of *comEA* by TfoX / CRP-cAMP complex and QstR (Figure 1).

### Narrowing down the promoter region driving the expression of *comEA*

**Figure 3:**
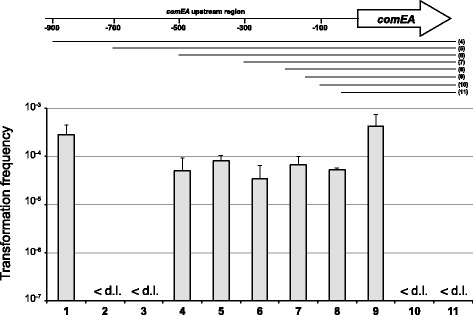
**Localization of the putative promoter region of** ***comEA*** **.** Schematic representation of *comEA* with 900 bp of its upstream region (not to scale). To localize the promoter, the upstream region of *comEA* was incrementally shortened. The numbers indicate the bp upstream of the start codon of the open reading frame, which itself is indicated by the arrow. The cloned fragments are indicated by the lines below the genomic region view, and the numbers in the brackets on the right correspond to the lane numbers in the graph. Fragment lengths upstream of *comEA* (numbered 4–11): 900 bp, 700 bp, 500 bp, 300 bp, 203 bp, 134 bp, 100 bp, 40 bp. Graph: *V. cholerae* strains harboring the plasmid-encoded *comEA* plus the indicated upstream regions were tested for their transformability. The transformation assay was performed in a chitin-dependent manner, and the transformation frequencies of the strains are shown on the y-axis. Strains tested: wild-type A1552 (lane 1); ΔcomEA (lane 2); ΔcomEA/p (vector control; lane 3) and ΔcomEA containing plasmids according to the schematic above the graph (lanes 4–11). The data are the average of at least three independent experiments and the error bars reflect the standard deviation. <d.l.: below detection limit.

To better understand how the expression of *comEA* is regulated*,* we first mapped the putative promoter region of this gene. To do so we constructed eight plasmids carrying the gene and its upstream region, which we incrementally shortened (Figure 3). The plasmids were originated using direct or inverse PCR and plasmid pBR-[own]*comEA* as template [22]; Table 1). This plasmid contains the *comEA* gene and a 900 bp sequence that is upstream of the start codon and therefore includes the promoter region [22]. All eight constructs were tested for their ability to restore the transformability of the *V. cholerae comEA-*minus strain (∆comEA) *in trans* after chitin-dependent induction of competence (Figure 3). ∆comEA strains harboring plasmid-encoded *comEA* and at least 134 bp of its upstream region were complemented and showed transformation frequencies comparable to the wild-type strain. Neither of the bacterial strains harboring either 100 bp or 40 bp of the *comEA* upstream region rescued transformability, nor did the strain carrying the empty vector (Figure 3). We concluded that 134 bp of sequence upstream of the *comEA* start codon are sufficient to drive *comEA* expression.

### Prediction of putative promoter elements within the *comEA* upstream region

**Figure 4:**
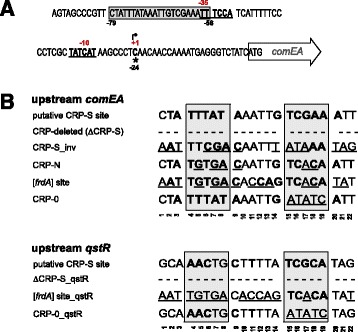
**The transcriptional start site of** ***comEA*** **and the putative CRP-S site. (A)** Sequence of the upstream region of *comEA.* Numbers in black below the nucleotide sequence indicate the position relative to the start codon (ATG, as indicated); numbers in red above the nucleotide sequence refer to the position relative to the transcription start site at position −24 (highlighted by the arrow and +1). The putative −10 and −35 regions are also highlighted in bold and underlined. The newly identified putative CRP-S site is centered at position −44.5 (sequence surrounded by the gray box). **(B)** Putative CRP-S sites identified in the upstream region of *comEA* and *qstR* and their site-directed mutagenesis. The conserved bases of the putative CRP-S site are indicated in bold (compared to the motif *in silico* predicted by Cameron and Redfield for other competence genes of *Vibrionaceae* [20]). The putative CRP-S site upstream of *comEA* and *qstR* was entirely deleted in the constructs ΔCRP-S and ΔCRP-S_qstR, respectively. Changes made to generate the indicated CRP-S motif variants are underlined.

Next, we localized the transcription start site of the *comEA* transcript using 5′ RACE (Rapid amplification of cDNA ends). As schematized in Figure 4A, we could map the transcription start site at −24 bp upstream the start codon. In *E. coli,* the majority of the promoters consist of two conserved hexanucleotides, which are located at approximately −35 and −10 from the transcription start site. The consensus of a “typical” promoter of *E. coli* has been summarized as follows: TTGACA-(N_15–19_)-TATAAT-(N_5–7_)-start [63] (N = any nucleotide). The −35 and −10 regions are specifically recognized and bound by the σ subunit of the RNA polymerase (RNAP) holoenzyme [64], and the most commonly used σ factor in *E. coli* is σ^70^ [65]. Comparing the sequence upstream of the transcription start site of *comEA* with the consensus of the *E. coli* σ^70^-activated promoter [65], we identified the putative −35 and −10 regions of the *comEA* promoter, **TT**TC**CA**-(N_16_)-**TAT**C**AT**-(N_7_)-start, as shown in Figure 4A. These regions were also predicted using the BPROM program, which works to recognize bacterial σ^70^ promoters [66]. Moreover, we manually screened the upstream region of *comEA* and identified a motif similar to the *in silico* predicted consensus of the *Vibrionaceae* CRP-S site [20] (Figure 4A and B). As described above, the CRP-S site is hypothesized to represent a CRP binding motif where the protein binds to in conjunction with TfoX. This putative CRP-S site is 22 bp in length and located between −79 bp and −58 bp upstream of the *comEA* start codon (Figure 4A).

### Investigation of the putative CRP-S site upstream of *comEA*

**Figure 5:**
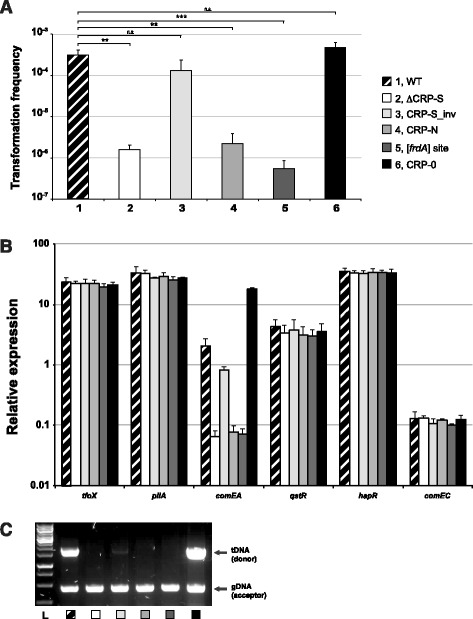
**Variants of the putative CRP-S motif preceding** ***comEA*** **are altered with respect to natural transformation. (A)** Natural transformability of the indicated strains was tested under *tfoX*-inducing but chitin-independent conditions as previously described [10]. The frequencies are indicated on the Y-axis. Lane numbers are according to the legend shown on the right. The graph shows the average of at least three independent biological replicates (±SD as indicated by the error bar). Statistically significant differences were determined by Student’s *t-*tests*.* **P < 0.01, ***P < 0.001, *n.s.* = not significant. **(B)** The same strains used in panel A were tested for the relative expression of *tfoX* (arabinose-induced), *pilA, comEA, qstR, hapR*, and *comEC*. The values are given on the Y-axis. Data are averages from at least three independent experiments ± SD. **(C)** The ability of the wild-type and mutant *V. cholerae* strains to take up DNA was tested using a whole-cell duplex PCR assay. The lower fragments reflect the quantity of acceptor bacteria and serve as internal controls. The upper band indicates internalized transforming DNA (tDNA). L, ladder.

To understand the importance of this putative CRP-S site, we modified its sequence using site-directed mutagenesis at the original chromosomal locus. The sequences of the putative CRP-S site upstream of *comEA,* as well the site-directly changed CRP sites are represented in Figure 4B. Specifically, we either deleted the entire putative CRP-S site (ΔCRP-S), inverted it (CRP-S_inv), changed it to the consensus of the canonical CRP binding site, which works independently of TfoX (CRP-N), or to the *in silico* predicted CRP-N motif of the *V. cholerae* fumarate reductase gene [20] ([*frdA*] site), or we altered it to a motif that differed considerably from the original CRP-S consensus in the 3′ half of the motif (designated CRP-0) (Figure 4B). When we tested these modified strains, we observed a perfect correlation between natural transformability (Figure 5A) and the expression levels of *comEA* (Figure 5B). The (non-) functionality of the CRP-S motifs in driving *comEA* expression was also confirmed by testing DNA uptake using a recently developed whole-cell duplex PCR assay [9,12,13] (Figure 5C). Notably, all of these assays confirmed that the absence of the CRP-S site lowered the *comEA* expression level followed by reductions in ComEA-mediated DNA uptake ability and eventual transformation (Figure 5). Interestingly, the mutant carrying the inverted CRP-S site turned out to be only mildly impaired in transformation (Figure 5A) due to reduced expression of *comEA* (Figure 5B) and reduced DNA uptake (Figure 5C). Most importantly, the replacement of the CRP-S site by both CRP-N derivatives (CRP-N and [*frdA*] site) resulted in similar phenotypes as those seen with the CRP-S-minus strain. The CPR-0 motif mutant on the other hand displayed the highest expression level for *comEA*, which resulted in increased DNA uptake and transformation (Figure 5).

### The CRP-S site upstream of *qstR*

**Figure 6:**
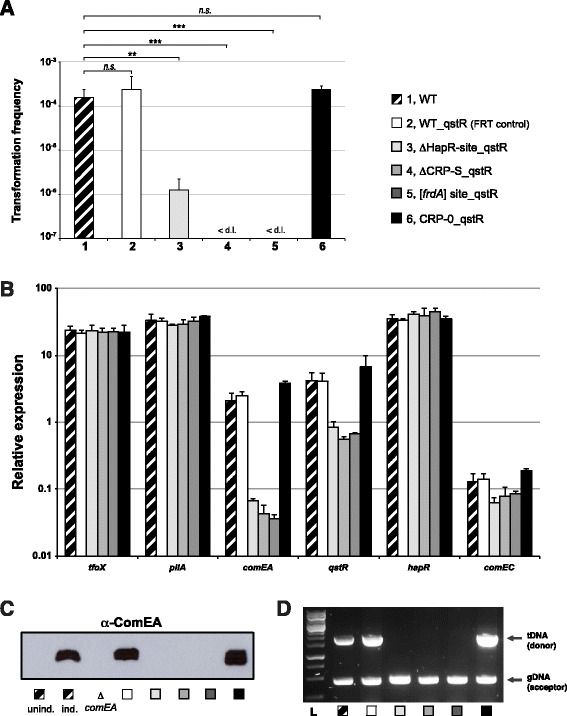
**Altering the promoter region of** ***qstR*** **affects natural transformation.** Variants lacking either the HapR-binding site or the putative CRP-S site or containing site-directly changed CRP-S motifs were tested for natural transformability, relative expression of the selected genes, ComEA protein production, and DNA uptake **(panels A-D)** as described for the previous figures. The tested strains are indicated in the legend, and all experiments were performed at least three independent times. Error bars represent the standard deviation. Statistically significant differences with respect to natural transformation were determined by Student’s *t-*tests*.* **P < 0.01, ***P < 0.001, *n.s.* = not significant. The value of the detection limit was used for the statistics of non-transformable strains.

From the data presented above, we hypothesized that the putative CRP-S motif indeed plays a role in the regulation of *comEA*, but potentially not in the same way that would be expected from a *bona fide* CRP-S site. Notable, expression of *comEA* also requires QstR, which links the QS pathway with the competence genes [22]. Because *qstR* expression occurs in a TfoX-dependent manner [22], we speculated that the gene might also be preceded by a putative CRP-S site. Indeed, upon closer inspection we identified such a motif in the upstream region of *qstR* (−87 bp to −65 bp upstream from the ATG start codon of the ORF) (Figure 4). To investigate the contribution of this site to *qstR* expression and natural transformability, we either deleted the putative CRP-S motif or used site-directed modifications as indicated in Figure 4. All of these variants were chromosomally-encoded and replaced the original putative CRP-S site. As a control for the genetic engineering method that we used to exchange the motif (TransFLP; [53,55,60], we also included a strain in which the FRT scar preceding the putative *qstR* promoter region was present without an altered CRP-S motif. This strain (WT_qstR (FRT control)) showed WT behavior with respect to all measured phenotypes (e.g., expression of *qstR*, natural transformation, and DNA uptake; Figure 6). Moreover, we also included a strain in which the *in vitro*-identified HapR-binding site [22] was deleted (strain ΔHapR-site_qstR). Notably, this strain was severely impaired in transformation due to very low *qstR* and *comEA* expression, undetectable ComEA protein levels, and a DNA uptake ability, which was below the detection limit of the assay (Figure 6). With respect to the putative CRP-S site, we observed similar phenotypes as those observed in the mutants that were modified in the upstream region of the *comEA* gene (described above). That is, both deleting the motif and changing it to an *frdA*-derived CRP-N site ([*frdA*] site_qstR) abolished *qstR* and *comEA* expression, ComEA production, DNA uptake and, consequently, natural transformability. Remarkably, the change to a CRP-0 site again slightly exaggerated those same phenotypes (Figure 6).

## Discussion

In bacteria, gene transcription begins only after 1) binding of the RNA polymerase (RNAP) holoenzyme to the promoter region and 2) formation of an open complex of the DNA. Promoters that are non-constitutively active (e.g., those of the competence genes) require one or several activating protein(s), such as the CRP protein, that directly interact with the RNAP and assist the holoenzyme in the steps preceding transcriptional initiation [67]. Using *comEA* and *qstR* as important genes of the competence regulon of *V. cholerae*, this study contributes to the understanding of the regulatory network driving natural competence. For *comEA* we showed that the region up to 134 bp upstream of the start codon is sufficient to drive *comEA* expression in this organism. But what initiates transcription of *comEA*? Trying to answer this question, we followed a common assumption, namely that the expression of the competence genes of *V. cholerae* is TfoX- and CRP-cAMP-dependent and that competence genes are preceeded by so-called CRP-S sites as previously suggested for *H. influenzae* [48,49]. And indeed, we demonstrated that the deletion of a newly identified motif with striking resemblance to the *in silico* predicted CRP-S sites [20] had a negative effect on natural transformability. Moreover, we also identified and investigated a putative CRP-S site upstream *qstR*, which likewise was required for *qstR* expression and natural transformability. Interestingly, our site-directed mutagenesis approach resulted in unexpected but interesting phenotypes. CRP-mediated activation at the CRP-S site is expected to be different from that at the canonical CRP binding site (CRP-N site; Sxy/TfoX-independent). We therefore assumed that exchanging the putative CRP-S site for a CRP-N motif would enhance competence gene expression. Notably, and in contrast to this assumption, the expression of *comEA* and *qstR* was significantly reduced when preceeded by a CRP-N site (Figures 5 and 6). Moreover, opposite results were observed for the strains carrying the CRP-0 motif variants. Despite the fact that this mutation affects the most highly conserved bases of the *in silico* predicted CRP-S consensus (those in the 3′ part of the motif), the *comEA/qstR* genes were expressed at higher levels, which correlated well with increased levels of ComEA protein, enhanced DNA uptake, and higher transformation frequencies (Figures 5 and 6). We therefore speculate that the CRP-0 mutation could either be a better binding site for the RNAP or favor the escape of the RNAP complex from the promoter in order to begin transcription. However, based on these unexpected phenotypes we concluded that the identified motifs indeed play a role in driving the expression of *qstR* and *comEA* but that they do not qualify as *bona fide* CRP-S sites.

Upon visual inspection we did not identify other motifs resembling the CRP-S site within the putative promoter region of *comEA.* A sequence (TGCGA-N6-AAGCA) centered at −115.5 from the transcription start point and located between −147 bp and −132 bp upstream the *comEA* start codon, was recently discussed (though never experimentally addressed) by Antonova *et al*. The authors suggested that this sequence serves as a potential CRE element (competence regulatory element [49]; the former name for CRP-S sites) [68]. Although we cannot exclude that this sequence is indeed a CRP binding motif, this site is not essential for the transcription of *comEA*, as it was not part of the construct that *in trans* complemented the respective knockout strain (e.g. *comEA* preceded by 134 bp of its upstream region; Figure 3). Furthermore, the localization of this putative CRP-S site within the open reading frame of the adjacent gene (*VC1918*) also leads to questions regarding its functionality with respect to the regulation of *comEA*.

## Conclusion

Given the absence of any obvious alternative CRP-S site within the 134 bp upstream of *comEA* we raised the possibility that TfoX and CRP-cAMP only indirectly regulate *comEA* via the intermediate transcriptional regulator QstR. Indeed, we have previously shown that QstR is necessary for the expression of *comEA* and *comEC* but not of the DNA-uptake pilus-encoding genes [12,22]. However, upon artificial induction of *qstR in trans* only low levels of *comEA* transcript were measured in accordance with the production of low levels of the ComEA protein (Figure 2). Two hypothesis are therefore possible: either the proteins TfoX and/or CRP-cAMP are somehow involved in the production of the previously suggested co-factor of QstR [22], or a direct regulation of *comEA* by TfoX/CRP-cAMP still occurs but involves a CRP-S binding site that significantly differs form the consensus that was *in silico* predicted for the *Vibrionaceae* [20]. Further studies involving chromatin immunoprecipitation followed by high-throughput DNA sequencing (ChIP-seq) will be required to ultimately establish the *in vivo* binding sites of CRP-cAMP, QstR, and potentially also of TfoX in naturally competent *V. cholerae* cells.
